# Ending Intimate Partner Violence after pregnancy: Findings from a community-based longitudinal study in Nicaragua

**DOI:** 10.1186/1471-2458-9-350

**Published:** 2009-09-18

**Authors:** Mariano Salazar, Eliette Valladares, Ann Öhman, Ulf Högberg

**Affiliations:** 1 Centre for Demography and Health Research, Nicaraguan National Autonomous University, León, Nicaragua; 2 Department of Obstetrics and Gynaecology, Nicaraguan National Autonomous University, León, Nicaragua; 3 Epidemiology and Public Health Sciences, Department of Public Health and Clinical Medicine, Umeå University, Sweden; 4 Obstetrics and Gynaecology, Department of Clinical Sciences, Umeå University, Sweden; 5 Umeå Centre for Gender Studies, Challenging Gender Excellence, Umeå University, Umeå, Sweden

## Abstract

**Background:**

Although reducing intimate partner violence (IPV) is a pervasive public health problem, few longitudinal studies in developing countries have assessed ways to end such abuse. To this end, this paper aims to analyze individual, family, community and societal factors that facilitate reducing IPV.

**Methods:**

A longitudinal population-based study was conducted in León, Nicaragua at a demographic surveillance site. Women (n = 478) who were pregnant between 2002 and 2003 were interviewed, and 398 were found at follow-up, 2007. Partner abuse was measured using the WHO Multi-country study on women's health and domestic violence questionnaire. Women's socio demographic variables, perceived emotional distress, partner control, social resources, women's norms and attitudes towards IPV and help-seeking behaviours were also assessed. Ending of abuse was defined as having experienced any abuse in a lifetime or during pregnancy but not at follow-up. Crude and adjusted odds ratios were applied.

**Results:**

Of the women exposed to lifetime or pregnancy IPV, 59% reported that their abuse ended. This finding took place in a context of a substantial shift in women's normative attitudes towards not tolerating abuse. At the family level, no or diminishing partner control [OR_adj _6.7 (95%CI 3.5-13)] was associated with ending of abuse. At the societal level, high or improved social resources [OR_adj _2.0 (95%CI 1.1.-3.7)] were also associated with the end of abuse.

**Conclusion:**

A considerable proportion of women reported end of violence. This might be related to a favourable change in women's norms and attitudes toward gender roles and violence and a more positive attitude towards interventions from people outside their family to end abuse. Maintaining and improving social resources and decreasing partner control and isolation are key interventions to ending abuse. Abuse inquiring may also play an important role in this process and must include health care provider's training and a referral system to be more effective. Interventions at the community level are crucial to reducing partner violence.

## Background

While Intimate Partner Violence (IPV) figures are usually underreported and comparison between studies is difficult due to different abuse measurements[1], IPV is very common. In a review of population-based studies around the world, Heise et al. [2]reported a 10-50% lifetime physical abuse prevalence. Recent findings from ten countries with data collected using a standardized instrument described a physical or sexual IPV lifetime prevalence varying from 15% to 71%[3]. Figures from Nicaragua ranged from 40% in general to 52% for partnered/married women[4].

Continuous abuse is frequent; however, rates differ due to different follow-up periods[5]. Data from community-based studies with a follow-up time between two to three years reported an abuse continuation rate between 50 and 76% [6-8]. In contrast, a community-based study with a longer follow-up period (6 years) reported a lower re-abuse rate (16%)[9]. Continuation of abuse has also been assessed on women using health services. A three year-long longitudinal study with rural women enrolled in a primary care screening program in the United States reported a 37% re-abuse rate[10]. In Nicaragua, one study found that 25% of IPV victims would leave an abusive relationship after four years, and this increased to 75% after 22 years[11].

Few longitudinal studies have analyzed factors that are associated with persistent IPV. Several individual risk factors for continuous abuse were reported: women of older age [10], previous physical or sexual abuse in adulthood[6], poor social support, high psychological distress[12], time couple has lived together[13] and number of dependants[9]. Partner's risk factors included low socioeconomic status and high levels of marital conflict[13].

In addition, recent cross-sectional data from 10 countries reported that women experiencing physical or sexual IPV were more likely to be controlled by their partners[3]. Although individual and relationship factors are important, the relevance of cultural norms and values justifying men's violence against women also has been highlighted[1].

In contrast, significant social support[14,15], employment, and the availability of people in their networks who provided help were related to ending abuse[15]. Furthermore, gender training combined with microfinance intervention has also been described as helpful. Results from a cluster randomized trial in South Africa showed that women who received the intervention had 55% IPV reduction at the end of the two year follow-up period[16].

The health sector might have played an important role in ending abuse by diminishing isolation[17] and improving community connectedness[18]. The American Medical Association recommends that health care providers routinely ask patients about IPV[19]. In fact, most women agree to IPV screening [20-22]. Many women believe that such screening could be useful[20]. However, the feasibility of routinely asking about IPV screening in resource-poor settings has been questioned because of lack of confidentiality and adequate referral services[19]. Screening, however, may provide women with information about resources in their communities and improve their mental health by allowing them to disclose their IPV experiences.

This study is a follow-up of previous research conducted at this setting on the magnitude and characteristics of IPV during pregnancy[23]. Although there is controversy whether pregnancy is a protective or risk factor for partner abuse[24], it is clear that women experiencing partner abuse before or during pregnancy can also continue to experience abuse in the future. Hence, when studying factors related to abuse cessation, it is important to depart from life-time and during pregnancy exposure. To the best of our knowledge, there are no longitudinal studies in low-income countries that assess how to end abuse determinants or that ask women about their experiences with violence. To address this need, this paper aims to analyze individual, family, community and societal determinants for ending abuse. In addition, we explore women's experiences of ending abuse in relation with IPV inquiry.

## Methods

### Study setting and procedures

A longitudinal study was conducted in León municipality, Nicaragua. Pregnant women were selected from León Health and Demographic Surveillance System baseline 2002-2003 (HDSS). It covers 50 randomly selected geographical clusters representing 22% of the total municipality population [25].

Initially, 483 women were identified as pregnant by a screening question posted during HDSS baseline data collection. Four women were excluded: two were identified not to be pregnant and two households contained two pregnant women living in the same house. Due to the sensitivity of the issue, only one woman per household was interviewed. One woman declined to join the study [23]. Therefore, 478 pregnant women were assessed for IPV experiences before and during pregnancy and reassessed three years later in the first four months of 2007.

At follow-up, 83% (398) of the women were found. The main reason for drop out was migration. One woman died from cervical cancer and one declined to be interviewed. Those 80 women lost to follow-up did not differ from women contacted regarding age, education, residency, parity, emotional or physical/sexual IPV, social resources, and partner control prevalence at baseline.

The questionnaire for the WHO Multi-country study on women's health and domestic violence (Sections 6-9) was used to measure IPV magnitude and characteristics[3]. IPV was defined as being exposed to emotional, physical or sexual violence. At baseline, IPV was measured regarding lifetime and during pregnancy exposure. At follow-up, it was assessed whether abuse had occurred in the previous twelve months before the interview. Hence, ending of abuse was defined as having been exposed to IPV in a lifetime or during pregnancy, but not at follow-up, three years later.

Violence against women ecological framework[26] was employed as a guide to study variables associated with ending abuse at different levels. At the individual level, women's age, marital status, employment, education, parity, residency and perceived emotional distress (measured by the Self-Report Questionnaire, SRQ 20)[27] were assessed. At the family level, partner control was explored using section 7 of the WHO Multi-country study on women health and domestic violence questionnaire. It consisted of seven items that describe how a woman's most recent partner controls her behaviours and activities[3]. It was further dichotomized into two options: no controlling behaviours or one to seven behaviours.

A community-level variable measured was socioeconomic status. It was calculated by a method validated in Nicaragua, the Unsatisfied Based Needs Assessment [28]. At the societal level, social resources, gender-related attitudes toward help-seeking behaviours and women's norms and attitudes towards IPV were collected. Social resources were assessed by a method developed by Hanson et al[29] and modified to be used during pregnancy by Dejin-Karlsson et al[30]. It consisted of an index evaluating three major concepts: social network (evaluating social anchorage and social participation), social support (measuring emotional, instrumental, father of the child, maternal and parental support) and sense of control of daily life. Items within each scale were added and dichotomized to the lowest tertile, which was defined as low. The same procedure was applied to the index's scores. The lowest tertile was defined as low social resources.

Section 6 of the WHO Multi-country study on women health and domestic violence questionnaire was used to study women's gender-related attitudes toward help-seeking behaviours and norms and attitudes towards IPV[31]. Gender-related attitudes toward help-seeking behaviours were explored measuring the level of agreement to the following sentence: *If the husband mistreats the wife, other people from outside the family should intervene*. Norms and attitudes towards IPV were measured using a six-item question that asked the participants what reasons would make it appropriate for a man to abuse his partner.

The possible effect of previously asking about IPV was explored with the following question: *"Did it make a difference when we asked you about violence when we visited you three years ago?"*.

Two trained female interviewers collected the data. A field supervisor reviewed all instruments on site. Questionnaires with inconsistencies were returned to the field for rectification.

### Analysis

Prevalence was calculated with 95% CI. Age at follow-up was stratified by its quartile distribution. In order to assess the effect that changing temporal patterns of social resources and partner control had on ending abuse, each variable's exposure during pregnancy and exposure during follow-up were combined into one variable. Combined social resources were dichotomized into two options: high at both time points or increased and low at both time points or decreased. Combined partner control also had two options: high (women controlled at both time points) or increased and no control at both time points or decreased.

Crude and adjusted ORs with 95% CIs were calculated with SPSS-15 statistical package (SPSS, Chicago IL). Only women reporting ever in life or at pregnancy IPV (229) were included in the multivariate analysis. Age and variables with p-value < 0.05 were included in the model.

### Ethics

The study protocol was approved by the Ethics Research Committee of León University, Nicaragua. WHO ethical guidelines for research on domestic violence[32] were followed. Written inform consent was obtained from participants. Free referral services were available for women who needed them.

## Results

### IPV temporal patterns and prevalence

**Figure 1:**
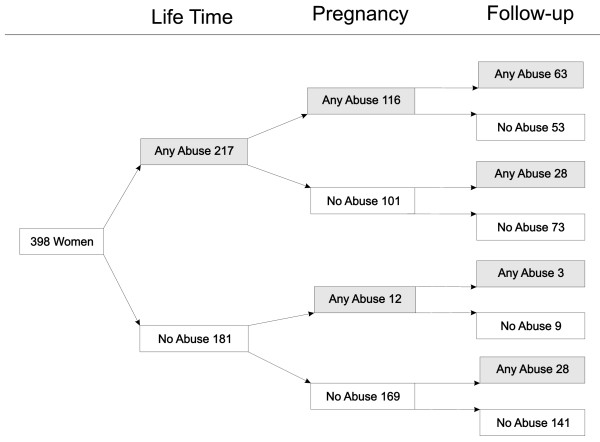
**IPV patterns when women were contacted at follow-up**. A community-based longitudinal study (2002-2007), León, Nicaragua (n = 398)

The results show that two-thirds of the women (257/398) had been exposed to violence at some time - lifetime, during pregnancy, or at follow-up. About half of the women who at baseline reported lifetime violence exposure-53% (116/217) - were also exposed during pregnancy. Of those being exposed during pregnancy, half of the women - 51% (66/128) - reported violence exposure at follow-up (Figure 1).

Prevalence of violence was the same during pregnancy as at follow-up, 32% and 31%. This prevalence was because 28 women had their first exposure to violence during follow-up (Figure 1). They were mainly urban (82%), highly controlled at follow-up (75%), with the same pregnancy partner (75%), and unemployed (64%). Of the women who experienced any lifetime IPV or IPV during pregnancy, 59% (52-65% CI) reported no abuse at follow-up (135/229).

### Ending of abuse determinants and perceived emotional distress

Women ending abuse were significantly more likely to have a new partner, to be alone at follow-up, or to live in a rural area. Perceived emotional stress at follow-up was significantly lower for women ending abuse. We observed a decline in partner control of women's activities and social resources from baseline to follow-up. Furthermore, no partner control and high social resources were significantly associated with abuse ending at both time points. Time combined partner control and social resource analysis revealed that women who ended violence had higher prevalence of improved or high social resources at both pregnancy and at follow-up three years later than women who presented continuous violence. In addition, they showed higher prevalence of decreased or no partner controls at both pregnancy and at follow-up three years later (Table 1).

**Table 1:** Women's baseline and follow-up characteristics by IPV patterns (continued abuse vs. ending and never abused)*, n = 370.

**Characteristics**	**Level**	**Continued abuse** **n = 94**		**Ending abuse** **n = 135**		**Never abused** **n = 141**	
		**n**	**(%)**	**n**	**(%)**	**n**	**(%)**
Women's age at follow-up (years)	18-23	31	(33)	36	(27)	39	(28)
	24-27	29	(31)	36	(27)	36	(25)
	28-31	19	(20)	31	(23)	27	(19)
	32-50	15	(16)	32	(23)	39	(28)

Marital status baseline	Partner	86	(91)	113	(84)	112	(79)
	Alone	8	(9)	22	(16)	29	(21)†

Marital status follow-up	Same partner	82	(87)	101	(75)	112	(79)
	New partner/alone	12	(13)	34	(25)†	29	(21)

Employment follow-up	Yes	32	(34)	41	(30)	50	(36)
	No	62	(66)	94	(70)	91	(64)

Women's education	> 3rd grade	38	(40)	65	(48)	95	(67)
	≤ 3rd grade	56	(60)	70	(52)	46	(33)†

Parity	1	25	(27)	44	(33)	69	(49)
	2 or more	69	(73)	91	(67)	72	(51)†

Residency	Rural	32	(34)	68	(50)	22	(16)
	Urban	62	(66)	67	(50)†	119	(84)†

Socioeconomic status	Non poor	36	(38)	46	(34)	71	(51)
	Poor	58	(62)	89	(66)	70	(49)

Perceived emotional distress baseline	SRQ ≤ 6	41	(44)	70	(52)	110	(78)
	SRQ ≥ 7	53	(56)	65	(48)	31	(22)†

Perceived emotional distress follow-up	SRQ ≤ 6	47	(50)	97	(72)	118	(84)
	SRQ ≥ 7	47	(50)	38	(28)†	23	(16)†

Partner control baseline	None	17	(18)	58	(43)	89	(63)
	1-7 activities	77	(82)	77	(57)†	52	(37)†

Partner control Follow-up	None	20	(21)	90	(67)	109	(77)
	1-7 activities	74	(79)	45	(33)†	32	(23)†

S. resources baseline	High	44	(47)	88	(65)	102	(72)
	Low	50	(53)	47	(35)†	39	(28)†

S. resources Follow-up	High	33	(35)	70	(52)	76	(54)
	Low	61	(65)	65	(48)†	65	(46)†

Combined partner control	High-increased	74	(79)	45	(33)	32	(23)
	No control-decreased	20	(21)	90	(67)†	109	(77)†

Combined social resources	Low-decreased	61	(65)	65	(48)	65	(46)
	High-increased	33	(35)	70	(52)†	76	(54)†

*Women who experienced abused only at follow-up (28) not included, † p < 0.05

### Women's norms and attitudes toward gender roles

Ending of abuse was found in a context where women's norms and attitudes toward gender roles and violence changed significantly over time. At follow-up, women were significantly more likely to express positive attitudes regarding the intervention of people outside their family to end abuse. In addition, a significant decrease in women's attitudes toward societal norms justifying violence was found. These attitudinal changes remained also for women never reporting IPV (Table 2).

### Perceived effect of abuse inquiring at baseline on ending of abuse   

Of the women previously exposed to violence (229), 41 (18%) answered that to be asked about abuse at baseline helped them in their process to be free of partner abuse.

**Table 2:** Women's attitudes toward gender roles between time points and by IPV patterns at Follow-up, n = 398.

	**Between time points**				**% at follow-up**					
**Percentage of women answering " yes" to following statements**	**Baseline** **n = 398**	**Follow-up** **n = 398**	%difference*		**Continued abuse** **n = 94**		**Ending abuse** **n = 135**		**Never abused** **n = 141**	
	**%**	**%**	**%**	**95%CI**	**%**	**95%CI**	**%**	**95%CI**	**%**	**95%CI**
If husband mistreats wife, other people outside family should intervene.	55	67	12	(5;19)	70	(60-80)	70	(62-78)	64	(55-72)
A man has good reasons to hit his wife if:										
1. Doesn't do house chores	6	2	-4	(-6;-1)	2	(0-7)	3	(1-7)	1	(0-4)
2. Disobeys husband	7	3	-4	(-6;-1)	4	(1-1)	4	(1-8)	1	(0-4)
3. Doesn't want to have sex	2	1	-1	(-2;0)	1	(0-6)	---		---	
4. Asks him about another woman	3	1	-2	(-3;0)	2	(0-7)	---		---	
5. Suspects she is unfaithful	8	1	-7	(-10;-4)	3	(1-9)	1	(0-4)	---	
6. Discovers she is unfaithful	13	1	-12	(-15;-8)	2	(0-7)	---		---	

*McNemar Test

### Crude and adjusted analysis

Crude Odds Ratios showed that rural residency, being with a new partner or alone at follow-up, a decreased or no partner control at both time points, and an increase or high social resources at both time points were determinants for IPV cessation. Multivariate logistic regression revealed that combined partner control [Or_adj _6.7 (95%CI 3.5-13)] and social resources [Or_adj _2.0 (95%CI 1.1.-3.7)] remained significant after adjustment for age, residency, and marital status at follow-up (Table 3).

**Table 3:** Abuse ending, crude and adjusted OR by selected baseline and follow-up determinants, n = 229.

**Characteristics**	**Level**	**Crude OR**	**95% CI**	**Adjusted OR**	**95% CI**
Age at follow-up (years)	18-23	1		1	
	24-27	1.06	(0.5-2.1)	0.9	(0.4-1.9)
	28-31	1.4	(0.7-3)	1.6	(0.7-4)
	32-50	1.8	(0.8-4)	2.1	(0.8-5.4)

Residency	Urban	1.0		1.0	
	Rural	1.9	(1.1-3.3)	1.8	(0.9-3.4)

Marital status follow- up	Same partner	1.0		1.0	
	New partner/alone	2.3	(1.1-4.7)	1.9	(0.8-4.5)

Combined Partner control baseline and follow-up	High-increased	1.0		1.0	
	Low-decreased	7.4	(4-13.6)	6.7	(3.5-13)

Combined Social Resources baseline and follow-up	Low-decreased	1.0		1.0	
	High-increased	2.0	(1.1-3.4)	2.0	(1.1-3.7)

## Discussion

This study demonstrates that determinants at two levels in the violence against women ecological framework were associated with patterns of ending abuse. At the societal level, a normative change was observed during a period of three years; a steep decrease in women's norms and attitudes tolerating abuse were found whether abused or not. Moreover, ending abuse was associated with high or improved social resources. At the family level, no or diminishing partner control on women's activities was also related. Although abuse prevalence at pregnancy and at follow-up remains unchanged, women are ending abuse faster than previously reported in this setting[11].

The results of this study are consistent with previous finding reporting that high social support is a protective factor for women exposed to violence by men[33,34]. Our longitudinal results demonstrate that maintaining or improving women's social resources is a key way to ending abuse. Furthermore, high social resources can play an important role in the process of recovering women's health after the abuse has ended[35].

To the best of our knowledge, this study is the first longitudinal study that shows that keeping low or diminishing partner control on women's activities is paramount to ending abuse because it decreases isolation, encouraging women to seek help from varied resources. Community interventions that support men's behavioural change by challenging controlling masculinity as the norm in society[19] might help diminish partner control.

Because community tolerance to gender violence has been described as a significant factor for partner abuse[34], one key finding in this study is that women at follow-up express remarkably fewer norms and values that support abuse than at baseline irrespective of their experience with IPV. Moreover, they are more likely to express positive attitudes regarding the intervention of people outside their family to end partner violence.

This positive change might be influenced by numerous interventions conducted since 1990 by the Nicaraguan women's movements and several NGOs that have focused on legislation change, psychological/legal counselling, and media campaigns challenging the cultural norms that define gender roles and partner abuse[36,37]. From these results, we cannot directly assess attitude change on the general population; however, recent evidence - an evaluation of a national mass media intervention that includes our study site - supports these findings[37].

Another significant finding is that one-fifth of the previously abused women found abuse inquiry helpful in ceasing or diminishing partner violence. This is in line with evidence from other settings that show that screening might reduce violence[17] by reducing isolation[18]. Also, screening or abuse inquiring at health facilities can improve community connectedness by referring abused women to specialized non-health services in their communities [38]. However, IPV screening is a controversial matter. Women re-victimization can be an issue in facilities where the personnel lack training[38] and where there are few adequate referral sources[19]. Nevertheless, others see it as an opportunity for health prevention [39], recognizing the benefits even when only conducting routine inquiry versus screening[40]. Further research is needed to evaluate advantages and disadvantages in resource poor settings.

### Strengths and limitations

This study was conducted as a follow-up of pregnant women, so the results can be generalized to ending of abuse after pregnancy. However, since we followed all pregnant women from a representative sample of the population (DHSS)[25], we believe that women contacted at follow-up might represent all mothers in the study site. External validity is strengthened because there was no statistical difference between drops outs and women contacted at follow-up. During data collection, validating and using the same instrument at baseline and follow-up minimized systematic errors. In addition, in order to diminish information bias, researchers in charge of data collection and interviewers at follow-up were blinded to women's original abuse status. Causality in relationship to changes in marital status, partner control and social resources must be examined with caution because we cannot assess whether these changes at follow-up occurred before or after abuse ended. In addition, since the prevalence of outcome is high, odds ratios might overestimate the associations.

## Conclusion

IPV continues to be a paramount problem for Nicaraguan women; however, a considerable proportion of women reported ending abuse. After decades of community-based and media campaign interventions, it is clear that there has been a substantial decrease in women's norms and attitudes tolerating abuse and a more positive attitude toward interventions from outside the family to end violence against women. Because these normative changes might be related to ending abuse, they must be a key component in any program addressing IPV in societies where a high tolerance to violence against women by men is found. In addition, the data provided here suggest that maintaining and improving social resources and decreasing partner control and isolation are key interventions that can help end abuse. Abuse inquiry may also play an important role in this process by diminishing isolation.

In many countries, including Nicaragua, the burden of ending partner abuse has been placed on non-governmental organizations. Although this is a great effort, it is clearly not enough. Thus, it is paramount that governmental policies and interventions are created in order to reach more women in need. This can be more achievable in settings where medical and public health services are free and widely used, such as in Nicaragua. This approach will allow the inclusion of abuse inquiry as a part of routine services offered to women. It might help end abuse by diminishing isolation, providing information and improving social resources.

## Competing interests

The authors declare that they have no competing interests.

## Authors' contributions

MS and EV participated in the design of the study, as principal investigators, in recruiting women into the study, were responsible for the data collection, contributed to the analysis and helped to write the report. AÖ participated in the analysis and helped to write the report. UH participated in the design of the study, as principal investigator, contributed to the analysis and helped to write the report. All authors read and approved the final manuscript.

## Pre-publication history

The pre-publication history for this paper can be accessed here:


